# Drug Reaction With Eosinophilia and Systemic Symptoms Syndrome in a Patient Taking Lamotrigine: A Case Report Based Literature Review

**DOI:** 10.7759/cureus.22359

**Published:** 2022-02-18

**Authors:** Zahid Khan, Vinod Warrier, Syed Aun Muhammad, Animesh Gupta, Yousif Yousif, Ayub Khan, Abdullah Afghan, Donatas Taucius, Mohammed Abumedian, Maab Ibrahim, Mohammed Mohammed, Gideon Mlawa

**Affiliations:** 1 Cardiology, Royal Free Hospital NHS Foundation Trust, London, GBR; 2 Internal Medicine, Mid and South Essex NHS Foundation Trust, Southend-on-Sea, GBR; 3 Cardiology, Mid and South Essex NHS Foundation Trust, Southend-on-Sea, GBR; 4 Acute Internal Medicine, Barking, Havering and Redbridge University Hospitals NHS Trust, London, GBR; 5 Internal Medicine, Barking, Havering and Redbridge University Hospitals NHS Trust, London, GBR; 6 Emergency Medicine, Barking, Havering and Redbridge University Hospitals NHS Trust, London, GBR; 7 Internal Medicine, Daisy Hill Acute Hospital, Newry, IRL; 8 Gastroenterology, Mid and South Essex NHS Foundation Trust, Southend-on-Sea, GBR; 9 Geriatrics, Barking, Havering and Redbridge University Hospitals NHS Trust, London, GBR; 10 Internal Medicine/Haematology, Sandwell and West Birmingham NHS Trust, Birmingham, GBR; 11 Internal Medicine and Diabetes and Endocrinology, Barking, Havering and Redbridge University Hospitals NHS Trust, London, GBR

**Keywords:** drug-induced urticarial rash, drug-induced hepatitis, international normalized ratio, international normalized, high fever, systemic steroids, generalized rash, allergy test, allergy and anaphylaxis

## Abstract

A 29-year-old patient presented to the hospital with worsening generalized rash for the last two days from a mental health facility. The patient was commenced on lamotrigine two weeks earlier, and he developed fever and generalized macular rash on his body. His blood tests showed deranged liver function tests (LFTs) and clotting with raised eosinophil count, and he was treated for lamotrigine-induced drug reaction with eosinophilia and systemic symptoms (DRESS) syndrome. The patient was commenced on prednisolone 50 mg once daily with a proton pump inhibitor cover, and lamotrigine was suspended on advice from Dermatology. The patient showed improvement after 3-4 days of treatment. His skin biopsy showed prominent suppurative granulomatous folliculitis, mild perivascular chronic inflammation, and red blood cell extravasation, including the rare eosinophil. He was weaned off from prednisolone by 5 mg weekly and had complete resolution of symptoms.

## Introduction

Drug reaction with eosinophilia and systemic symptoms (DRESS) syndrome is a delayed hypersensitivity reaction (DHR) that presents because of a severe drug reaction or offending agent [1]. DHR includes DRESS syndrome, Stevens-Johnson syndrome (SJS), vasculitis, and maculopapular eruptions, although it may also present as simple, generalized, or localized rash [2]. Patients tend to develop fever, diffuse popular, macular, pustular, or vesicular rash once the disease process starts [2]. In addition to skin manifestation, hypersensitivity reaction may also have hematologic manifestations such as lymphadenopathy, thrombocytopenia, and/or lymphocytosis. It is worth mentioning that eosinophilia is not universally present even though the disease has been named “drug-induced hypersensitivity syndrome” [3].

Hypersensitivity reactions can occur with both common and novel drugs. It can affect any organ and may present from simple allergic rash to potentially life-threatening anaphylaxis [2, 4]. The offending drug can be stopped in most cases, although in some instances, it may need to be continued in the absence of a suitable alternative, in which case desensitization may be required.

The task force of the European Academy of Allergy and Clinical Immunology and the American Academy of Asthma Allergy and Immunology has classified DHRs into allergic and non-allergic categories [4]. The DHRs happen either through immunoglobulin E (IgE)-mediated mast cell activation or non-IgE-mediated mast cell activation, and anaphylaxis usually happens through the activation of mast cells. Another classification for the DHRs is based on the time of onset of symptoms and are classified into immediate reactions (IgE-mediated response) and non-immediate hypersensitivity reactions (T-cell mediated response) [4]. The incubation period of DRESS syndrome is about 2-8 weeks, and one proposed mechanism for symptoms emergence is the reactivation of human herpesvirus 6 (HHV-6). However, the exact mechanism for this HHV 6 reactivation remains unclear. One proposed etiology described is that the offending drug or its metabolites might contribute to the T-cell induced viral reactivation [5]. Research has shown that diuretics and anticonvulsants are associated with an increased risk of DRESS syndrome, and genetic factors seem to play an essential role in this [5]. In addition, other drugs associated with DRESS syndrome include several antiepileptics, antibiotics, and sulphonamide drugs such as dapsone, sulfasalazine, and sulfamethoxazole [6, 7].

## Case presentation

We present a case of a 29-year-old patient who was transferred to our hospital from a mental health unit where he was under treatment for extreme depression and mental health issues. His only significant medical history includes depression, previous suicidal attempts, and bipolar disorder. His medications include mirtazapine and quetiapine, and he was commenced on lamotrigine 25 mg twice daily two weeks ago. He developed a generalized macular skin rash two weeks after commencing lamotrigine followed by a high-grade fever of 38.5 C. The rash started initially with mild redness, followed by morbilliform eruption and blisters on his body. There was evidence of mucosal involvement in lips only. He had mild non-tender cervical lymphadenopathy on examination.

His liver screen, including viral hepatitis screen, cytomegalovirus (CMV), Epstein-Barr virus (EBV), HHV, anti-mitochondrial antibody, anti-smooth muscle antibody, antinuclear antibody, serum immunoglobulins, complement levels, 24 hours urine copper, serum copper, caeruloplasmin, alpha antitrypsin, and mycoplasma screen were negative. His hepatitis B core antibody test was positive, but the hepatitis B surface antigen was negative.

The patient was diagnosed with DRESS syndrome secondary to recently introduced lamotrigine, and all his regular medications were suspended temporarily. This diagnosis was based on the raised eosinophil count, skin involvement, deranged liver functions suggesting systemic involvement, deranged clotting, and normal liver and autoimmune screen, as shown in Table 1. His symptoms started within two weeks of starting the offending medication (lamotrigine in this case), and his symptoms persisted for further 4-5 weeks, which is the usual course for DRESS syndrome. In addition, this diagnosis was supported by the fact that the patient had a fever, systemic symptoms, maculopapular rash, raised eosinophil count, lymphadenopathy, and negative viral hepatitis screen. His symptoms started to show improvement after 2-3 weeks which is consistent with the findings in DRESS syndrome.

**Table 1:** Trend of lab results for the patient.

Blood test	Normal value	Day 1	Day 7	Day 28
White cell count	(4.0-11.0) 109/L	7.6	6.2	5.3
Neutrophil	(1.7-7.5) 109/L	6.5	6.0	5.5
Eosinophils	(0.0-0.4) 109/L	1.8	0.90	0.42
Platelet	(150-400) 109/L	186	172	232
Sodium	(133-146) mmol/L	136	138	137
Potassium	(3.5-5.3) mmol/L	4.5	4.6	4.5
Urea	(2.5-7.8) mmol/L	1.5	4.5	3.2
Creatinine	(59-135) umol/L	61	79	55
International Normalized Ratio (INR)	(0.8-1.2) INR	1.7	1.5	1.1
Alkaline phosphatase	(30-130) U/L	45	40	30
Total bilirubin	(0-21) umol/L	55	48	22
Conjugated bilirubin	(<3.4) umol/L	38	36	18
Alanine transaminase (ALT)	(<50) U/L	1788	1588	55
Amylase	(28-100) U/L	54	62	45
Paracetamol	<15 mg/L	<15	<10	Undetectable
Prothrombin time (PT)	(10.3-13.3) seconds	20.7	19.8	12.8
Ammonia	(16-53) umol/L	90	75	40
Lactate dehydrogenase (LDH)	240-480) U/L	1606	995	420
Gamma GT	(<55) U/L	455	235	45
C-reactive protein	(<5) mg/L	153	95	8
Aspartate aminotransferase (AST)	(<50) U/L	791	507	45
Lactate dehydrogenase (LDH)	(240-480) U/L	1606	567	340
Caeruloplasmin	(0.20-0.45) g/L	0.23		
Copper (serum)	(12.0-25.0) umol/L	18.7		

Skin biopsies showed prominent chronic suppurative granulomatous inflammation, and the superficial dermis showed mild perivascular chronic inflammation together with RBCs extravasation and rare eosinophils. The special stain for fungi was negative. Ultrasound (US) abdomen showed non-complicated small gall stones and fatty liver. Chest radiograph was clear, and urine midstream specimen was negative.

He was treated conservatively with IV fluids (IVF) and antihistamine. The dermatology team reviewed the patient and started him on oral prednisolone 50 mg once in the morning (OM) and took skin biopsies from his chest. Over the course of admission, the patient's INR normalized to 1.1, and liver function tests (LFTs) improved. The patient was advised to continue with oral prednisolone 50 mg OM along with proton pump inhibitors and Adcal-D3 (combination of calcium and vitamin D3) one tablet twice daily until dermatology OP in two weeks. The reason for Adcal-D3 was the prolonged course of steroids, and it did not have any role in the management of DRESS syndrome. The patient was advised to stay off his medications for the next one month and reintroduce mirtazapine and quetiapine following that, and lamotrigine was stopped. The patient showed significant improvement and his blood tests normalized. In addition, his rash showed improvement and completely disappeared after a month of treatment when he was seen in the outpatient clinic during follow-up visit.

## Discussion

DRESS syndrome is a severe, type IV DHR to certain drugs, particularly anticonvulsant medications, and is often underdiagnosed [8, 9, 10]. It was first described in 1936 due to an allergic reaction to an anticonvulsant drug [8,9]. DRESS syndrome usually presents with fever, generalized body rash, and raised eosinophil count; however, the most prominent feature is a systemic response in the form of deranged LFTs, kidney functions, or other major organs [11, 12].

The pathogenesis of DRESS syndrome remains unclear, and it is believed to occur because of a complex interplay between a patient’s genetic predispositions, abnormalities in metabolic pathways resulting in reduced elimination of drug metabolites, and viral interactions leading to reactivation of HHV-6 and HHV-7, EBV, and CMV which are responsible for this syndrome [13, 14]. Studies have shown that certain human leukocyte antigen (HLA) groups in some ethnic populations are associated with the development of DRESS syndrome after exposure to certain medications [15, 16]. It has been reported that minocycline is associated with a higher prevalence of DRESS syndrome in Afro-Caribbean blacks. In contrast, HLA-B*5701-associated abacavir-induced DRESS syndrome and HLA-B* 5801-associated allopurinol-induced DRESS syndrome are more prevalent in certain Chinese groups [17, 18].

It can be challenging to diagnose DRESS syndrome without ruling out other etiologies as several other diseases can also present with fever, multisystem involvement, and skin rashes. These disorders include infectious causes such as viral exanthemas, staphylococcal and streptococcal shock syndromes, meningococcaemia; non-infectious drug eruptions such as SJS, toxic epidermal necrolysis (TEN); autoimmune diseases such as Kawasaki disease, Still's disease, hypereosinophilic syndrome; and neoplastic diseases such as leukaemia cutis and mycosis fungoides [19]. The likely diagnosis would also depend on the organ involved, for example, viral hepatitis in case of liver involvement, glomerulonephritis in case of renal involvement, and eosinophilic myocarditis in case of cardiac involvement. Historically, the patient was exposed to causative drugs to confirm allergy. However, this should not be done in the case of DRESS syndrome due to the serious nature of this disease [19, 20]. It can be challenging to differentiate between DRESS syndrome, SJS, and TEN based on history only, as all three can present between 2 and 6 weeks after the commencement of the offending drug. However, in DRESS syndrome and SJS, the most common prodromal symptoms are itching, fever, and malaise. DRESS syndrome mainly involves the liver, whereas SJS mainly involves the mucosal membrane of the oral cavity and eyes. The most common causative agents in both diseases are antibiotics followed by anticonvulsants. In addition, patients with DRESS syndrome have prodromal symptoms of itching, fever, and facial edema, whereas patients with SJS commonly present with prodromal symptoms of fever and malaise. The first skin lesions appear on extremities and face in case of DRESS syndrome. In SJS, first skin lesions appear on the trunk, usually tender on palpation. The oral cavity, genitalia, and eyes are more commonly affected in SJS [21].

There is a lack of formal diagnostic criteria for DRESS syndrome; however, a Japanese working group established a set of diagnostic guidelines to diagnose patients with suspected DRESS syndrome as shown in Table 2 [8].

**Table 2:** Japanese working group criteria for DRESS syndrome. Source: [8]

Serial number	Diagnostic criteria
1	Maculopapular rash developing for more than three weeks after starting a drug
2	Prolonged clinical symptoms two weeks after discontinuation of the causative drug
3	Fever more than 38°C
4	Liver abnormalities (including ALT greater than 100 U/L)
5	Leukocyte abnormalities (either leukocytosis greater than 11×109/L, an atypical lymphocytosis, or eosinophilia greater than 1.5×109/L);
6	Lymphadenopathy
7	Human herpesvirus 6 (HHV-6) reactivation

The patient in our case report met at least six of these diagnostic criteria from 1 to 6. The patient described here met all of these described criteria for a diagnosis of DRESS except that his HHV-6 screening test was negative.

The Registry of Severe Cutaneous Adverse Reaction (RegiSCAR) is another scoring system developed for diagnosis for DRESS syndrome published in 2007, as shown in Table 3 [20, 22].

**Table 3:** Registry of Severe Cutaneous Adverse Reaction (RegiSCAR) criteria. Source: [20] ANA: Anti-nuclear antibodies.

Score value	Clinical findings/Lab values
1	Fever more than 38.5°C
1	Enlarged lymph nodes
1	Eosinophilia
1	Atypical lymphocytosis
1	Skin involvement
1	Organ involvement
1	Resolution greater than 15 days
1	Evaluation of other causes (ANA, blood cultures, serology for hepatitis A virus, hepatitis B virus, hepatitis C virus, and chlamydia and/or mycoplasma)

Based on these criteria, the diagnosis of DRESS syndrome is unlikely with a final score <2; DRESS syndrome is possible with a final score of 2-3; it is probable with a final score of 4-5, and it is confirmed with a final score >5. The patient in our case report had a final score of 6 except for atypical lymphocytosis and a negative autoimmune screen. 

There are no randomized controlled trials (RCTs) to study the effectiveness of the most suitable pharmacological therapy, and the evidence mainly comes from case reports and retrospective studies. Systemic steroids remain the cornerstone treatment for DRESS syndrome, and patients not responding to steroids may be considered for IV immunoglobulin (IVIG) and/or plasmapheresis [22, 23]. The long-term complications of the disease include autoimmune diseases such as thyroiditis, diabetes mellitus type I, systemic lupus erythematosus (SLE), systemic sclerosis, or adrenal insufficiency, which can happen several months after the initial presentation [23, 24].

## Conclusions

Our patient presented with fever and systemic symptoms two weeks after he was commenced on lamotrigine. This is the first case report to our knowledge to identify patients developing DRESS syndrome secondary to lamotrigine. It is essential to diagnose these patients timely, and prompt treatment should be initiated to avoid complications. These patients ideally should be followed up in a few months. These patients can develop late complications, and clinicians need to be aware of this. With prompt treatment, these patients usually show good recovery.
